# Note upon the Rabies Epidemic in Rochester, with a Report of a Verified Death from Hydrophobia1Read before the Pathological Society of Rochester, January 17, 1901.

**Published:** 1901-05

**Authors:** George W. Goler

**Affiliations:** Health Officer, Rochester, N. Y.


### NOTE UPON THE RABIES EPIDEMIC IN ROCHESTER, WITH A REPORT OF A VERIFIED DEATH FROM HYDROPHOBIA.[1]

By GEORGE W. GOLER, M. D.,
Health Officer, Rochester, N. Y.

ABOUT June 1, 1900, we began to receive reports of dogs dying of a strange disease, which had not before come under the observation of most of the veterinarians. The symptoms in these animals were briefly, change in temperament, loss of appetite, posterior paralysis, mouth drop, convulsions and death. An investigation was begun in each of these cases by inoculating portions of the medulla beneath the dura of rabbits, which almost uniformily produced death represented as characteristic of paralytic rabies in from two to eight weeks. After a number of these examinations had been made, and after the observation of a number of cases of dogs dying with typical symptoms of furious rabies, it was unhesitatingly announced by the health department that there was an epidemic of disease in dogs, which upon examination verified by the highest authorities in the country, was determined to be rabies.

The announcement on the part of the health department was received, as such announcements usually are, with jeers, and thenceforth more than six months was required to enlighten the public so that the necessary precautions might be taken to prevent the extension of the disease. From the beginning of cold weather, or about

1. Read before the Pathological Society of Rochester, January 17, 1901.

November 1st, up to the 1st of April, an average of more than a dog a day has been reported as rabid; a great many persons have been bitten, from fifteen to twenty people have been sent to the Pasteur Institute at New York City for preventive inoculation, and two persons have died of the disease. Letting the unverified case of the disease pass, I wish briefly to report the following case:

On or about December 15th, a "cur collie" ran amuck in the western part of the town, biting six people in this city and adjoining towns. The dog was secured after a chase, the jugular ganglion removed, submitted to the rapid test, and the persons bitten recommended to go to the Pasteur Institute of New York for preventive treatment. Three or more of the bitten persons went, but one of them, L. C., aged 46, refused to go. He complained of pain in the bite, and lightning pains extending up the arm from the seat of the bite upon the hand. On January 3d, rabbits inoculated from this animal died of paralytic rabies, and Cook was again invited to take preventive treatment. On or about February 9th, he began to have maniacal seizures which were attributed to alcoholism for the man had always been a drinker, and since his bite had been drinking heavily. In these seizures he jumped from his bed, rushed out doors and back again, got down on the floor and tore at the carpet, most of the time making strange guttural noises. He could drink but a teaspoonful of water at a time, and even this small quantity of fluid was always followed by a convulsive seizure. His temperature was 101°; pulse, 120, respiration rapid and labored; he presented all the evidences of mental distress, and died in one of many convulsive seizures on February 11th.

A post mortem examination revealed the characteristic "wet brain" of an alcoholic, there was an old tubercular deposit in both lungs, and the kidneys presented evidences of an interstitial nephritis. The jugular ganglion and a portion of the medulla were removed. The ganglion prepared after the method of Van Gehuchten and Nélis, which Ravenel has elaborated, showed the most beautiful characteristic changes of rabies, as found in the jugular ganglion by this method. Portions of the medulla inoculated into rabbits produced death with characteristic paralytic rabies in sixteen days. Portions of the medulla were sent to Dr. D. E. Salmon, Bureau of Animal Industry at Washington; Dr. V. A. Moore, of Cornell University, and Dr. M. P. Ravenel, of the University of Pennsylvania. Animals inoculated from Cook's medulla by these gentlemen produced characteristic paralytic rabies in each case. In some of the cases the experimental work was carried into the second and third generations. In Dr. Ravenel's work, he also found the characteristic rabid lesion in the ganglion, taken from one of the rabbits inoculated from Cook's brain.

Here, then, is presented a case in which a dog with the clinical symptoms of rabies is known to have bitten a man; the dog is proven to have rabies, both by the rapid test and by the inoculation of animals. The man dies on or about the forty-sixth day after the bite with the clinical symptoms of hydrophobia, his body is submitted to a post mortem examination, in his jugular ganglion are found those changes characteristic of rabies, his medulla is examined and there is found the rabic tubercle of Babès. Four different observers inoculated animals from his medulla, and all secure like results, these experiments being carried by at least one of the observers into the second or third generation. In at least one of the cases the characteristic changes are found in the ganglion of one or more of the rabbits. Could there be stronger clinical and scientific evidences of the death of this man from hydrophobia?

